# Effectiveness of ultrasound-guided peripheral mandibular nerve block: A systematic review

**DOI:** 10.1097/MD.0000000000042509

**Published:** 2025-05-16

**Authors:** Daniah M. Alhazmi, Fatima M. Jadu

**Affiliations:** a Oral Diagnostic Sciences Department, Division of Oral and Maxillofacial Radiology, Faculty of Dentistry, King Abdulaziz University, Jeddah, Saudi Arabia.

**Keywords:** inferior alveolar nerve block, pain control, ultrasound-guidance

## Abstract

**Background:**

The inferior alveolar nerve block (IANB) is commonly used in dentistry for pain management before and after dental procedures. Traditional nerve block techniques can sometimes be associated with complications such as anesthetic failure and nerve damage. Ultrasound-guided (USG) nerve blocks have emerged as a promising alternative. This systematic review aimed to evaluate the effectiveness of USG mandibular nerve blocks in pain control in adults.

**Methods:**

A systematic search of English literature was conducted using the PubMed, Web of Science, Scopus, Google Scholar, and Cochrane Library databases. Articles published between 2020 and September 2024 were selected based on a set of preestablished inclusion criteria. These articles were analyzed according to the PRISMA-2020 guidelines to seek evidence of effective pain management following USG IANB.

**Results:**

Seven articles including 193 patients and 176 ultrasound scans met the eligibility criteria. The included studies reported the effectiveness of USG mandibular nerve blocks by using various pain measurement scales. In addition, other indirect measures of pain control, such as maximum mouth opening, were used.

**Conclusion:**

This systematic review showed that USG IANB can effectively manage pain in adult patients undergoing specific dental surgical procedures including third molar extraction. Furthermore, it can provide relief for those suffering from chronic orofacial pain associated with conditions, such as temporomandibular disorders.

## 1. Introduction

Mandibular nerve block is one of the most frequently used techniques in dental clinics. It numbs the mandibular teeth and surrounding structures.^[1]^ The method’s success rate largely depends on the clinician’s knowledge of the mandibular foramen (MF) location, the point at which the inferior alveolar nerve (IAN) enters the mandible, where the anesthetic solution is administered.^[2]^ However, individual anatomical variations can occur at the foramen.^[3]^ Due to these variations, the failure rate of mandibular nerve block has been reported to range from 20% to 25%.^[4]^ As a result, precise localization of the IAN before delivering the anesthetic solution is crucial to ensure a successful nerve block.

Localization of the IAN is typically performed indirectly by identifying the MF in traditional 2-dimensional (2D) images.^[5]^ Panoramic radiographs is commonly used for this purpose.^[5,6]^ However, panoramic radiographs have certain limitations that may lead to inaccurate MF localization.^[7,8]^ Therefore, alternative imaging and visualization techniques have frequently been explored.

A peripheral nerve block is used to control pain when standard oral pain medications such as opioids fail to alleviate discomfort.^[9]^ Furthermore, this technique can reduce both the pain and side effects of general anesthesia, including nausea and respiratory depression, which may occur postoperatively.^[10]^ This procedure involves injecting a local anesthetic with a fine needle near the primary nerve, supplying the area from which the pain emanates.^[10]^ A guidance device, such as a fluoroscope or ultrasound (US), is essential for directing the needle to the target area.^[10]^

Recently, US imaging has been explored in dentistry for various applications including nerve visualization.^[11,12]^ Several studies have reported that real-time ultrasound-guided imaging offers optimal localization of the IAN, thus facilitating profound mandibular nerve block.^[13]^ However, only a few studies have evaluated the effectiveness of ultrasound-guided mandibular nerve block in pain management.^[14,15]^

Therefore, this systematic review aimed to evaluate the effectiveness of ultrasound-guided peripheral mandibular nerve block in pain management.

## 2. Methods

This systematic review was conducted following the Preferred Reporting Items for Systematic Reviews and Meta-Analyses (PRISMA) guidelines.^[16]^ The study protocol was registered in the PROSPERO International Registry for Systematic Reviews (ID number: CRD42024484922). As this study was a systematic review of previously published literature, ethical approval and informed consent were not required.

### 2.1. Literature search strategy

A comprehensive and systematic search of English literature was conducted using the PubMed, Cochrane Library, Web of Science, Scopus, and Google Scholar electronic databases. The search terms used were as follows: (((((Ultrasound-guided) OR (US-guided)) OR (Ultrasound-guidance)) OR (Ultrasound imaging)) OR (US guidance)) AND (mandibular nerve block) OR (IAN canal) AND (pain control). Reference lists were consulted for further identification of relevant articles. In this study, the terms mandibular nerve and IAN were used interchangeably, as they refer to blocks of the same nerve. The inclusion criteria for the articles were as follows: they must be original peer-reviewed articles, they must involve adult human participants, and they must report success in terms of the absence of pain. Given the novelty of this topic, several studies have been conducted, including case reports and clinical trials. The search was conducted at the Faculty of Dentistry, King Abdulaziz University and included studies published up to September 2024.

### 2.2. Process of data collection

**Figure 1.**
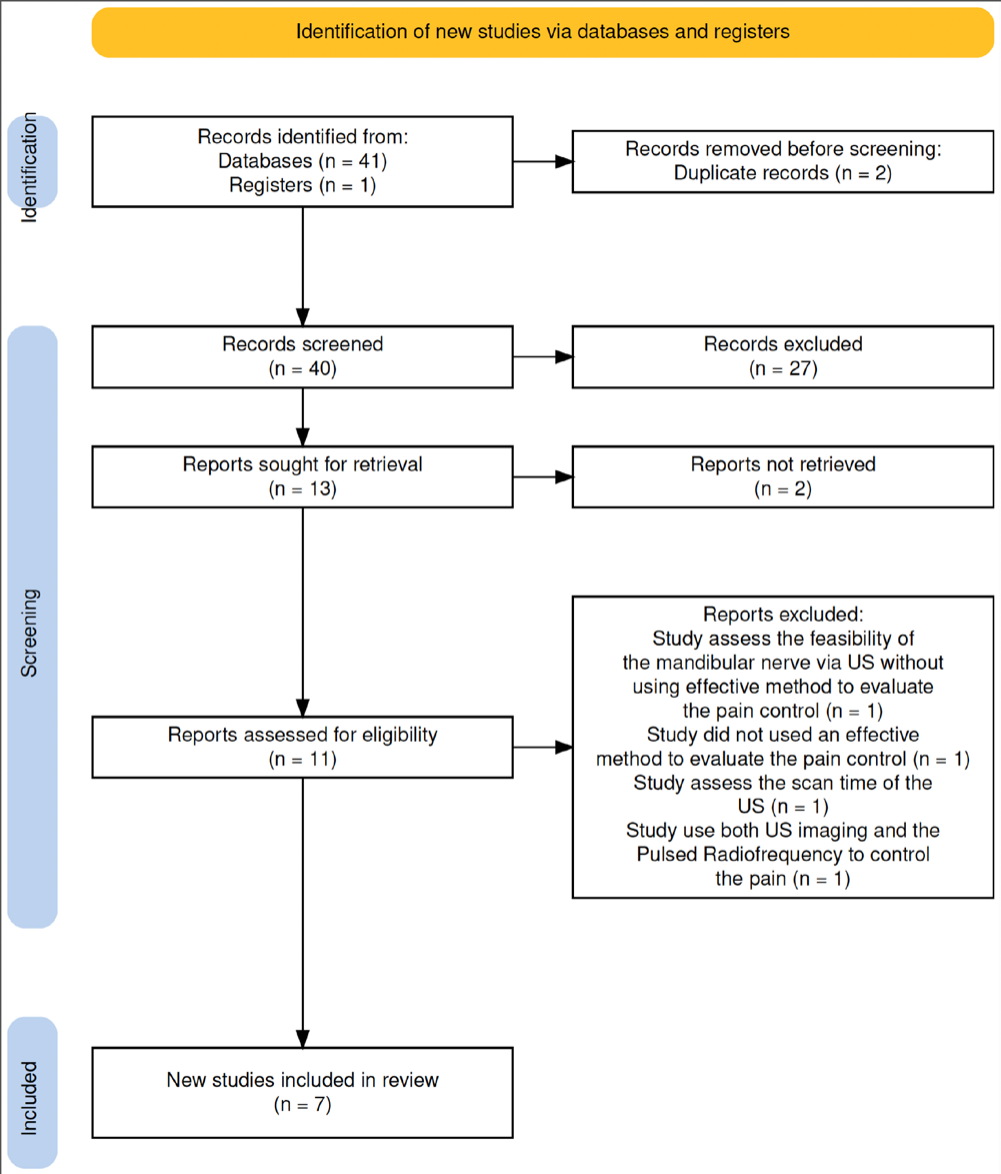
Diagram showing the selection of articles for the systemic review.

Two authors independently reviewed the selected studies and resolved all disagreements through consensus and discussion. The process of selecting studies was conducted in multiple stages, as illustrated in Figure 1. A flow diagram was developed following the PRISMA-2020 guidelines.^[17]^

### 2.3. Data extraction

The following data were extracted from each article: year of publication, sample size (number of patients and US imaging scans conducted), study design, technique used for nerve block, test used to measure pain control effectiveness (for instance, the visual analog scale [VAS]), and outcome (Table 1).

**Table 1:** Summary of the 6 articles included in the review.

Author and yr	Study design	Sample size	Technique	Results	Conclusion
Kojima et al 2020	Case report	1 patient 2 scans	USG IANB	• Pain (VAS score) reduced from 100/100 to 10 to 11/100 • Mouth opening improved from 20 to 40 mm	• Pain reduction • Mouth opening improvement
Jain et al 2020	Prospective, randomized controlled, outcome assessor blinded trial	32 patients 32 scans 32 patients	USG IANB VA-IANB	• Pain (VAS score) statistically significantly reduced for USG USG Preblock 64.37 ± 9.13 USG Postblock 3.21 ± 3.04 • Inter-incisal distance (trismus) statistically significantly increased for both groups USG Preblock 14.46±5.92 USG Postblock 39.14±4.23 VA Preblock 13.21±6.02 VA Postblock 38.65±4.20	• Pain reduction • Inter-incisal distance increase
Venkatraman et al 2021	Prospective, randomized, single-blinded trial	30 patients 30 scans 30 patients 30 scans	USG IANB before surgery (Group A) USG IANB after surgery (Group B)	• Total morphine consumption statistically significantly different Group A 4.566 ±0.717 mg Group B 5.93 ± 0.876 mg • Time to request a rescue analgesic significantly prolonged for group A Group A 794.08 ±89.561min Group B 505.33 ±73.159min • Intraoperative fentanyl consumption significantly reduced for Group A Group A 4 (13.3%) Group B 11 (36.7) • After the first 30 min, the heart rate was significantly less in Group A Group A 68.96 ±4.05 Group B 73.3 ±3.75 • VAS scores were significantly different after the 8h to 20h postoperatively Group A means 1.6 Group B means 2.27	• Morphine consumption reduced • Time to request rescue analgesic reduced • Intraoperative fentanyl consumption reduced • Better control of heart rate intraoperatively • Better pain scores
Kojima and Sendo 2021	Case report	1 patient 2 scans	Jaw manipulation using USG IANB (JMUI)	• Pain (VAS score) reduced from 100/100 to 21/100 • Mouth opening improved from 20 mm to 28 mm. • Anxiety (HADS) reduced from 24 to 9.	• Pain reduction • Mouth opening improvement • Anxiety reduction
Oiwa et al 2023	Case Report	6 patients 12 scans	USG IANB after extraction of bilateral mandibular third molars	• Pain NRS: POD 1 was 2/10 (mean) • QoR scale-40: POD 1 was 188.5 (mean)	postoperative pain after mandibular third molar extraction was effectively controlled.
Martinus et al 2024	Non-randomized prospective controlled study	18 patients 18 scans 18 patients	Study-USG-MNB Control-IANB	• VAS scores were significantly different during the IANB application USG-MNB median VAS score = 2 Control-IANB median VAS score = 4 • Prolonged the pain-free time after surgery USG-MNB 8h Control-IANB 4h • Reduced NSAIDs use after surgery USG-MNB 12 patients Control-IANB 4h 17 patients • USG-MNB achieved adequate surgical anesthesia	• USG-MNB successfully proved to be analgesic method for the lower third molar extraction surgery • Prolonged pain-free period and reduced the use of NSAIDs after lower third molar extraction
Esquerre et al 2024	Randomized control trial Prospective single blind	25 patients 50 scans 23 patients	USG V2 and V3 nerve block Intraoral infiltration	• Reduced cumulative OME consumption Day 1 Control 45.7 ± 37.6 mg Study 25.5 ± 19.8 mg Day 2 Control 64.5 ±60 mg Study 35.8 ± 30.2	Reduces postoperative opioid consumption by 50% in patient undergoing double-jaw orthognathic surgery.

Abbreviations: HADS = hospital anxiety and depression scale, MNB = mandibular nerve block, NRS = numerical rating scale (0–10), NSAIDs = nonsteroidal anti-inflammatory drugs, OME = oral morphine equivalent, POD = postoperatively day, QoR scale-40 = quality of recovery scale-40 score (40–200), USG = ultrasound-guided, USG IANB = ultrasound-guided inferior alveolar nerve block, VA-IANB = intraoral vazirani-akinosi-inferior alveolar nerve block, VAS = visual analog scale.

### 2.4. Outcome

The primary outcome evaluated was effectiveness of pain control. Common measures found in the literature include VAS, numerical rating scale (NRS), quality of recovery (QoR), and maximum mouth opening.^[18–20]^ The VAS is a pain rating scale evaluated on a 10-cm horizontal line where the left end represents “no pain” and the right end represents “worst pain.”^[18]^ The NRS is similar to the VAS but uses a numerical scale from 0 to 10 or 0 to 100, with zero signifying no pain and 10 or 100 indicating the worst pain.^[19]^ The QoR tool is a patient-based questionnaire consisting of several questions, one of which pertains to pain and is scored on an 11-point numerical scale.^[20]^ Maximal mouth opening measures the largest distance between the upper and lower anterior teeth using a measuring tape; reduced mouth opening typically suggests pain, and vice versa. All tools were evaluated at specified intervals, primarily before and after nerve block.

### 2.5. Analysis of the quality of included studies

All the selected studies were examined to identify any risk of bias. Two reviewers independently analyzed all the selected studies, and any disagreements were resolved through consensus.

**Figure 2.**
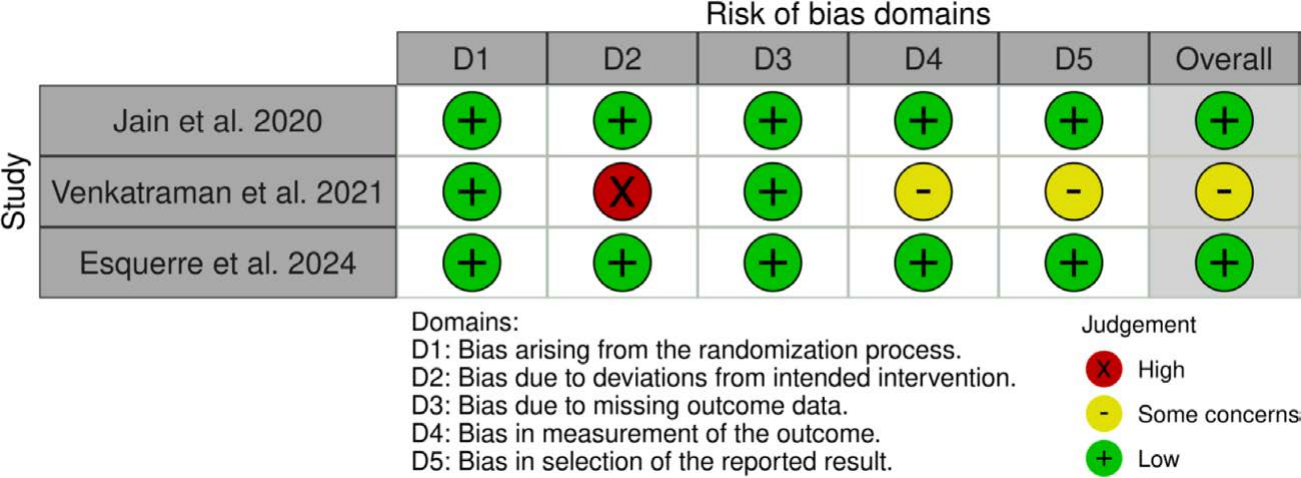
Risk-of-bias assessment for included randomized controlled trials using the RoB-2 tool. RoB = risk-of-bias.

**Figure 3.**
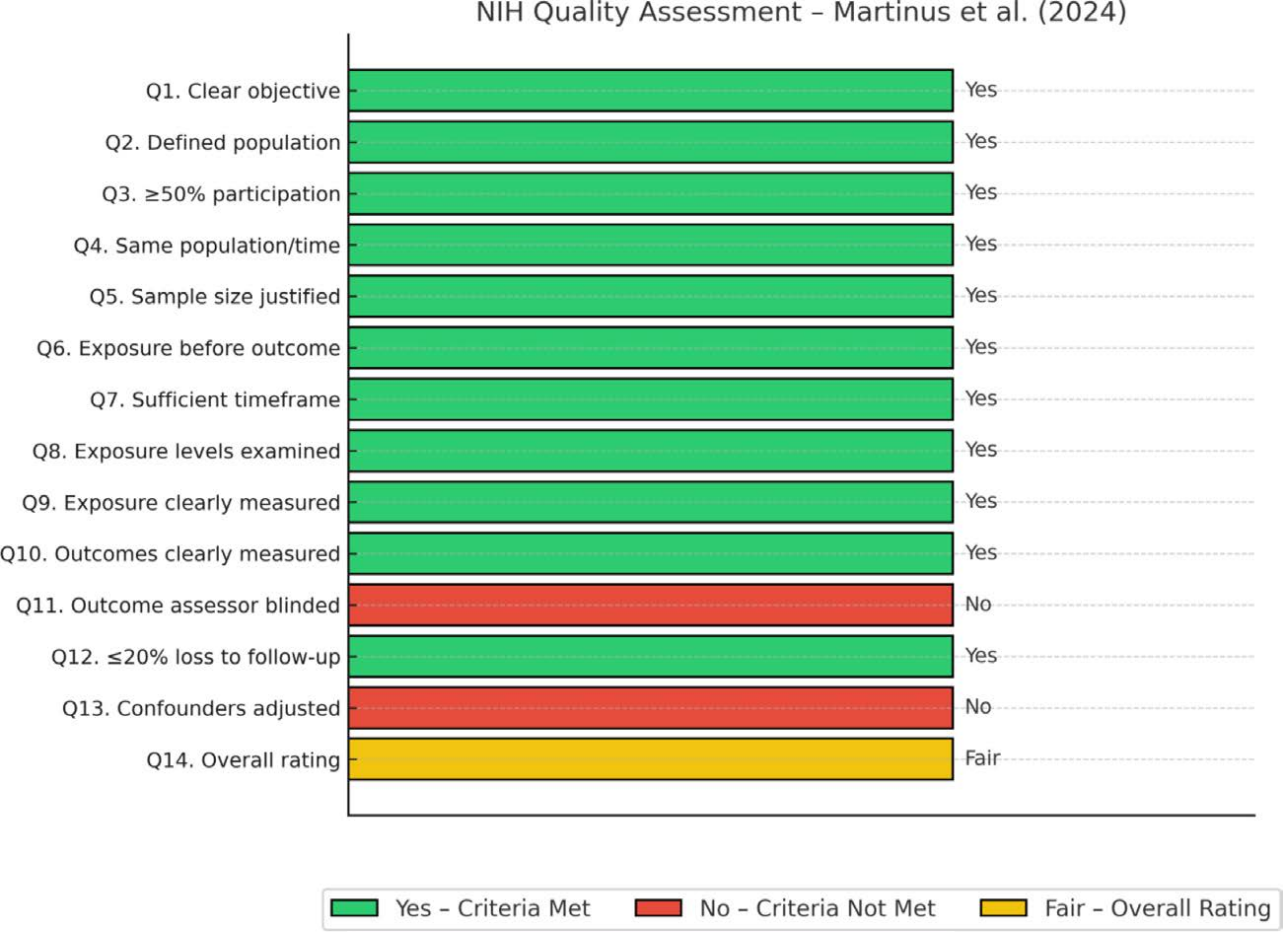
Summary of NIH Quality Assessment tool. NIH = National Institutes of Health.

**Figure 4.**
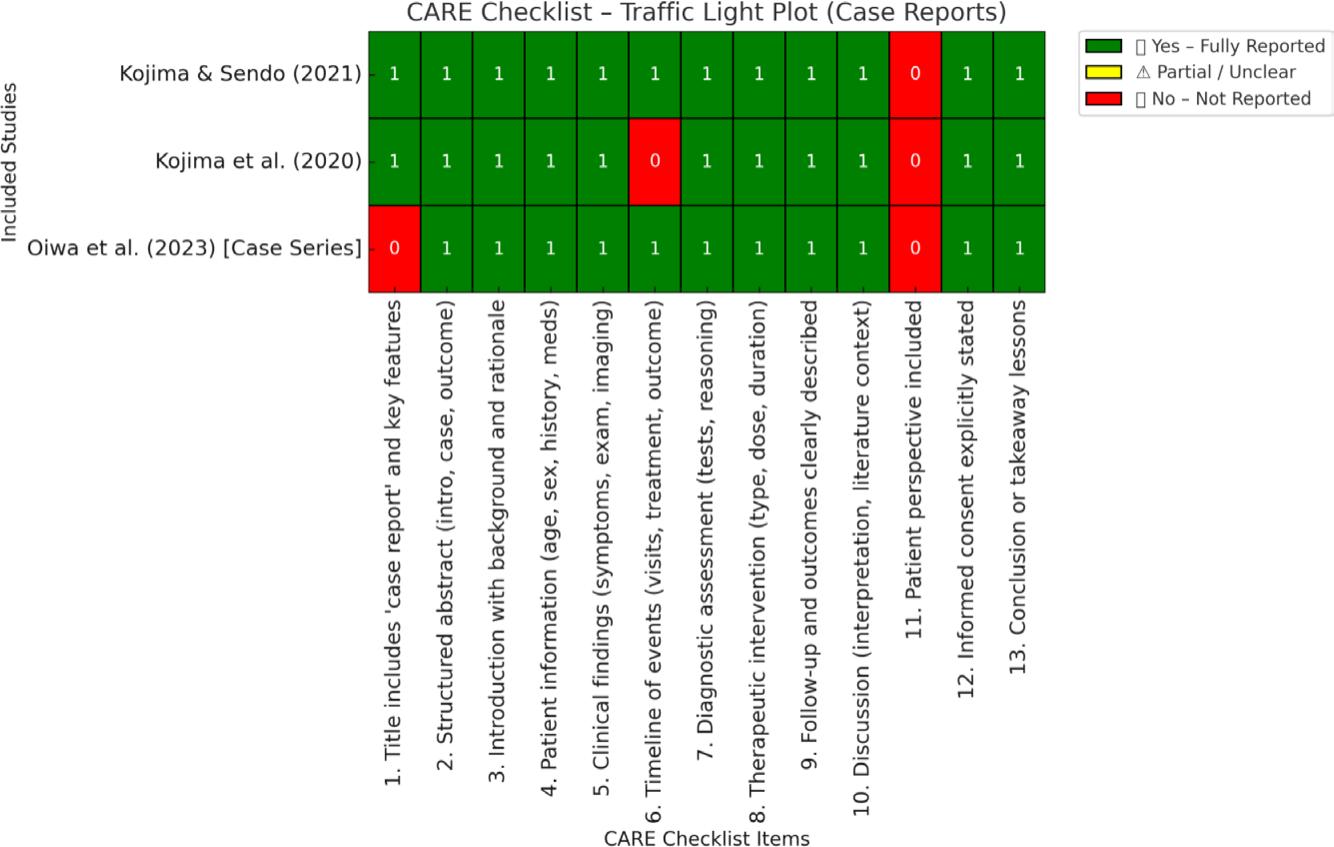
Case report checklist-based traffic light plot for included case reports.

As previously mentioned, this review includes several types of study design to provide a comprehensive overview of the topic. Therefore, we used various quality tests to assess the risk of bias. The revised Cochrane risk-of-bias tool for randomized trials (ROB-2)^[21]^ was used for the 3 randomized controlled trials, and the assessment results are presented in Figure 2.^[22]^ For the non-randomized controlled study by Martinus et al, we used the NIH tool for observational and cross-sectional studies.^[23]^ The results of this assessment are shown in Figure 3. Finally, we used the case report (CARE) tool^[24]^ to evaluate the quality of the case reports and case series. The results of this assessment are shown in Figure 4.

## 3. Results

### 3.1. Research and selection of studies

After reviewing the full text of the remaining 11 studies, 4 were excluded. One of these studies assessed the feasibility of visualizing the IAN using US imaging and measured the success rate using electric pulp testing (EPT), a dentist tool used to record the baseline vitality of teeth.^[13]^ The second study used the EPT as a tool to measure the success rate of nerve blocking using US imaging.^[14]^ The third study evaluated the scanning time required for US imaging when visualizing the IAN.^[25]^ In the fourth study, both US imaging and Pulsed Radiofrequency were used for pain management.^[26]^ Finally, 7 studies that met all the eligibility criteria were included in this review. The details are presented in Table 1.

### 3.2. Types of studies

Three of the 7 studies were randomized clinical trials.^[15,27,28]^ These 3 studies were single-blinded. The other study was a non-randomized, prospective, controlled study.^[29]^ Two others studies were case reports,^[30,31]^ and the final study was a case series,^[32]^(Table 1).

### 3.3. Sample sizes and characteristics

A total of 193 patients and 176 US scans were evaluated in 7 articles. The discrepancy in the number of patients and scans occurred because 4 of the studies had each patient undergo 2 US scans, 1 on each side of the mandible.^[28,30–32]^ In contrast, the remaining studies conducted only a single US scan for each patient in their study groups (Table 1).^[15,27,29]^

Two studies included patients with unilateral mandibular fractures,^[15,27]^ and 2 others included patients with severe trismus due to temporomandibular disorder (TMD).^[30,31]^ Two studies focused on controlling postoperative pain following the extraction of impacted bilateral mandibular third molars.^[29,32]^ One study involved patients in order to control postoperative pain after double-jaw orthognathic surgery.^[28]^

### 3.4. Evaluation of the US images

The included studies did not report on who performed or interpreted the US images.

### 3.5 Equipment

Only 5 of the 7 studies reported either the type of US machine or probe used (Table 2).^[15,27,29,32]^ Jain et al utilized a linear ultrasound probe (8–13 MHz [12 L-RS probe]; GE Healthcare LOGIQ e portable ultrasonography machine) and a cardiac probe (2.8–4 MHz [3S RS probe]; GE Healthcare LOGIQ e portable USG machine). Oiwa et al deployed a high-frequency linear probe (Venue Go; GE Healthcare), whereas Venkatraman et al employed a USG machine (Logiq V2; GE Medical Systems, Jiangsu, China) coupled with a 5 to 13 MHz linear probe. Martinus et al used a linear probe (Noblus US diagnostic scanner, 2019; Hitachi, Ltd.) with a frequency of 8 to 15 MHz. Esquerre et al utilized a US probe with an HFL50 of 15 to 6 MHz frequency. Details of the US types and parameters used in each study are listed in Table 2.

**Table 2:** Summary of the ultrasound-guided mandibular nerve/inferior alveolar nerve block approaches.

Author and year	Approach	Position of ultrasound probe	Information of the probe	Puncture point/inject point	Ultrasound-guided puncture method	Type and volume of the of anesthetic drug used
Kojima et al 2020	Extraoral	Not reported	Not reported	Not reported	Not reported	6 mL of 0.375% Ropivacaine on each side
Jain et al 2020	Extraoral	Superior to the mandible (linear ultrasound probe) Below the zygoma and anterior to the mandibular condyle (cardiac probe)	12 L-RS linear probe 8–13 MHz Cardiac probe 2.8–4 MHz	Superior (linear ultrasound probe) or posterior (cardiac probe) to the probe/Mandibular nerve	Out of plane (linear ultrasound probe) In-line (cardiac probe)	3 mL of 0.5% Bupivacaine
Venkatraman et al 2021	Extraoral	Below and parallel to the zygomatic arch	Logiq V2 linear probe 5–13 MHz	Cranial end/Maxillary artery at premandibular space	Out of plane	10 mL of 0.5% Ropivacaine
Kojima and Sendo 2021	Extraoral	Caudad to the zygomatic arch	Not reported	Superior Side of the probe/IAN at PS	Out of plane	6 mL of 0.375% Ropivacaine on each side
Oiwa et al 2023	Extraoral	transversely below the zygomatic arch	High-frequency linear probe	Cranial side of the probe/ IAN at PS	Out of plane	5 mL of 0.375% Levobupivacaine
Martinus et al 2024	Extraoral	Transversally onto the zygomatic arch at the top of the mandibular fossa	Noblus linear probe 8–15 MHz	Superior Side of the probe/ Mandibular nerve	Out of plane	2.5 mL of 0.75% Ropivacaine
Esquerre et al 2024	Extraoral	Infrazygomatic approach	HFL50 X 15–6 MHz	Superior Side of the probe/maxillary artery	Out of plane	5 mL of Ropivacaine

Abbreviations: IAN = inferior alveolar nerve, PS = pterygomandibular space.

### 3.6 Pain effectiveness measurements

All studies included either VAS or NRS to assess the effectiveness of pain control. Other metrics employed in the included studies to evaluate the analgesic effect of the ultrasound-guided (USG) mandibular nerve block included morphine consumption, duration of pain-free periods, and necessity for rescue analgesics. In 3 studies, maximum mouth opening was measured to gauge pain relief.^[15,27–32]^ Additionally, QoR was recorded in a study conducted by Oiwa et al. All tools were implemented at specific intervals, either before or after nerve block.

### 3.7. Outcomes

The primary outcome measure was the effectiveness of pain control. Two studies compared traditional mandibular nerve block with USG IAN block (IANB).^[15,29]^ Jain et al used the VAS score to compare pain reduction between the 2 techniques following unilateral mandibular fracture surgery. They found a statistically significant difference in the VAS scores for both techniques when comparing the scores before and after the blocks (*P*<.001). Pain was reduced after IANB with or without US guidance. However, they reported block failures (n = 6) in the traditional group and no block failures (n = 0) in the USG group.

Martinus et al used the VAS score to compare the pain levels following third molar extraction surgery between the 2 methods. The results demonstrated a statistically significant difference in VAS scores between the study and control groups (*P*<.001) during IANB. It was less painful to apply IANB with US guidance than it was without US guidance. Interestingly, there was no statistically significant difference in the VAS scores between the 2 groups when the pain level was measured at the time of administration of rescue analgesic medication 24 hours post-surgery (*P* = .597).

Conversely, Venkatraman et al compared the effectiveness of USG IANB for pain reduction both before and after mandibular fracture surgery. They found a statistically significant difference in the VAS scores between group A (those who used USG IANB after surgery) and group B (those who used USG IANB before surgery), from 8 to 20 hours postoperatively. The *P*-values were <.02, and <.03, respectively, indicating significant results, given that the *P*-value threshold was set at <.05. The study found a postoperative reduction in pain scores.

Postoperative morphine consumption was recorded in 2 studies.^[27,28]^ Venkatraman et al reported significant reductions in morphine consumption when an USG mandibular block was applied post-surgery (*P*<.0001). Additionally, Esquerre et al discovered that USG mandibular nerve block lowered the cumulative oral morphine equivalent (OME) consumption on both the first and second postoperative days (*P* = .023, = .40, respectively). The *P*-value was set at <.05.

Other assessed measures for pain control included the necessity for nonsteroidal anti-inflammatory drugs (NSAIDs) and the period until the demand for rescue analgesia. Martinus et al reported a decrease in the quantity of NSAIDs required after third molar extraction surgery (*P* = .038). In 2 separate studies by Venkatraman et al and Martinus et al, the amount of time before requesting rescue analgesics was noted. Both studies reported a significantly extended period before a request for rescue analgesics (*P*<.0001 and *P*<.001, respectively).

Venkatraman et al evaluated various parameters associated with the use of an USG mandibular block before and after unilateral mandibular fracture surgery. These parameters included intraoperative fentanyl consumption and heart rate during the initial 30 minutes. They found statistically significant reductions in intraoperative fentanyl consumption when USG mandibular block was administered before surgery. In addition, the heart rate was considerably lower, and the time until a request for rescue analgesia was significantly prolonged when USG was performed preoperatively (Table 1).

Oiwa et al, along with Esquerre et al, employed the NRS to evaluate postoperative pain control from the first to seventh days and on the first and second days, respectively. Significant differences between the first and seventh postoperative days have been reported by Oiwa et al Conversely, Esquerre et al did not observe any differences between the first and second postoperative days. Furthermore, Oiwa et al analyzed pain control using QoR tools. They observed significant differences in QoR scores between the first and seventh postoperative days.

Kojima et al and Kojima and Sendo conducted 2 studies using USG IANB to alleviate trismus and acute pain due to severe TMD. In both studies, assessments such as the VAS score and maximum mouth opening were evaluated before and after the procedure to determine the effectiveness of USG. The findings of these studies suggest that the application of USG IANB reduces pain levels and enhances mouth opening in patients. To evaluate trismus relief, Kojima and Sendo used the Hospital Anxiety and Depression Scale (HAD). They observed a significant enhancement in the HAD scores (Table 1).

Esquerre et al also evaluated the correlation between preoperative anxiety (measured by the Amsterdam preoperative anxiety and information scale) and postoperative pain, as reflected in the NRS and OME consumption on the second day after surgery. The results revealed a positive relationship between these 2 parameters. A synopsis of all studies included in our review is provided in Table 1.

### 4.4. Risk of bias in individual studies

This systematic review utilized the ROB-2 Cochrane tool for randomized controlled trials^[21]^ to assess the risk bias in 3 randomized clinical trials. Five domains were used to evaluate the risk of bias: bias arising from the randomization process, bias due to deviations from the intended interventions, bias due to missing outcome data, bias in the measurement of the outcome, and bias in the selection of the reported result. Each study was rated as having a low, some concerns, or a high risk of bias based on the overall assessment. Jain et al and Esquerre et al demonstrated a low risk of bias across all domains. However, Venkatraman et al reported a high risk of bias in the domain of bias owing to deviations from the intended intervention (Fig. 2).

Additionally, the risk of bias in a non-randomized prospective controlled study was evaluated using the National Institutes of Health tool (NIH).^[23]^ Clarity of objectives, methodological quality, validity of outcome measures, and control for confounding were ascertained through 14 questions. The study was categorized as good, fair, or poor, based on a comprehensive assessment. Martinus et al reported an overall rating of “fair” quality.’ This classification was attributed to the non-randomization of group allocation, absence of matching between the control and study groups, lack of blinding in the assessment of outcomes, and absence of statistical adjustment for potential confounders (Fig. 3).

Case reports and case series were evaluated using CARE.^[24]^ Thirteen domains were used to assess the completeness of the clinical case reports. All 3 case reports showed good-to-excellent results, as illustrated in Figure 4.

## 5. Discussion

Nerve block procedures, specifically those of the IAN, are frequently performed in dental clinics to manage pain prior to dental procedures.^[1]^ Conventional nerve block techniques may be associated with adverse effects such as failure to attain anesthesia and nerve injury.^[4,33]^ Recently, ultrasound-guided imaging has become increasingly prevalent in dentistry, especially in locating nerves prior to nerve block procedures.^[34]^ As a result, this systematic review aimed to examine the literature to assess the effectiveness of ultrasound-guided nerve blocks of the mandibular nerve in pain management.

This review included 7 studies, all of which confirmed that the use of USG IANB resulted in reduced pain in patients, irrespective of whether USG nerve block was administered before or after dental procedures for pain control. The dental procedures examined in these studies, such as mandibular fracture fixation surgery, were commonly associated with high levels of pain.^[27]^ The findings of this systematic review were consistent with those of another review that investigated the use of USG nerve blocks in the oral and maxillofacial regions.^[11]^ They also concluded that USG IANB was an effective analgesic.^[11]^

Three of the included studies also observed a significant improvement in mouth opening with USG IANB compared with the conventional nerve block technique.^[15,30,31]^ Furthermore, Venkatraman et al and Esquerre et al found that patients who received USG IANB required less morphine. In addition, Martinus et al noted a reduction in the number of NSAIDs required after a third molar extraction surgery. In 2 studies by Martinus et al and Venkatraman et al, patients experienced an elongated postoperative interval between requests for rescue analgesics.

Venkatraman et al also discovered that patients who underwent USG IANB experienced better control over their heart rate. The other findings examined in their study pertained to common complications often experienced after surgery under general anesthesia.

The 2021 study by Kojima and Sendo also collected HAD scores. This scale, which consists of 14 questions, was designed to assess mental health.^[35]^ Interestingly, these scores decreased after the use of USG nerve block to alleviate severe trismus caused by TMD.

In a study conducted by Martinus et al, the level of pain was assessed twice using VAS scores. Initially, the performance of the IANB was examined. Then, it was evaluated 24 hours after the third molar extraction surgery, particularly when the patients took the rescue analgesic. At the first assessment, the results echo those of other studies included in our analysis. However, the second review revealed no discernible differences between the control and the study groups. This disparity in results, notably at the second assessment, might stem from differences in the methodologies adopted for the outcome measurement. Martinus et al gauged the success of pain reduction using the VAS score only when patients required rescue analgesics. These methods were not congruent with those employed in other investigations that utilized VAS scores for all participants. Furthermore, patients were requested to self-report their pain levels 24 hours post-surgery. According to the NIH assessment tool, this self-reporting could be a potential source of bias influencing the study findings.

The risk of bias for the 3 clinical trial studies included in this review was assessed using the Cochrane Collaboration tool. Studies by Jain et al and Esquerre et al demonstrated a low risk of bias in all domains, indicating that our findings may be more robust and reliable than those of other studies.

Determining whether to perform a USG-imaging nerve block before or after surgery is a crucial part of the treatment planning. The 5 studies that were compared indeed reached the same conclusions. However, 4 studies used the USG IANB technique before surgery, whereas only one used it afterward. These studies concluded that using the USG technique to block the mandibular nerve before surgery not only reduced postoperative pain but also decreased morphine and intraoperative fentanyl consumption, prolonged the time before rescue analgesia was required postoperatively, and enabled better control of intraoperative heart rate.^[15,27–29]^ Although the Oiwa et al study had similar results as the previously mentioned studies, the results were not directly comparable due to the lack of a control group and the fact that only 6 patients were included.

There are 2 aspects tied to the clinical procedure of ultrasound-guided (USG) nerve block: the placement of the ultrasound probe and the plane of needle insertion in relation to the ultrasound probe. USG IANB can be performed either extraorally or intraorally.^[11]^ In all included studies, the extraoral approach was employed. This method was chosen mainly to enable the direct injection of anesthetic drugs into the pterygomandibular space.^[11]^ Moreover, some studies have provided detailed locations for extraoral probe placement. In the Kojima and Sendo studies, for example, the probe was set caudal to the zygomatic arch, whereas it was placed below and parallel to the zygomatic arch in both the Venkatraman et al and Oiwa et al studies. Interestingly, Jain et al used 2 US probes in their study: a linear probe placed above the mandible, and a cardiac probe positioned below the zygoma and anterior to the mandibular condyle. The needle insertion plane can be introduced either in-plane (parallel) or out of plane (perpendicular) to the probe. All included studies, except for Jain et al, used the perpendicular method of needle insertion owing to the use of 2 US probes, resulting in a needle insertion that was parallel to the cardiac probe but perpendicular to the linear probe.

Data on the type and volume of anesthetic drugs were not consistent across the 7 studies. On 1 hand, studies by Kojima and Sendo, Venkatraman et al, Martinus et al, Esquerre et al, and Kojima et al used the same anesthetic, ropivacaine, but with variable drug concentrations and volumes – specifically, 0.375%, 0.5%, 0.75%, with volumes ranging from 2.5 mL to 10 mL, respectively. In contrast, Jain et al used bupivacaine (3 ml of 0.5%), while Oiwa et al used 5 mL of 0.375% levobupivacaine. Table 2 presents the details of the anesthetic drugs used in each study.

Given the burgeoning practice of USG IANB, some limitations were observed in this systematic review. These issues primarily revolve around the methodological aspects of the studies involved. For instance, the study designs ranged from simple case reports to randomized controlled trials. However, these randomized controlled trials, as noted in the literature, had limited sample sizes. This review was restricted to the USG nerve block of the mandibular nerve, but the reasons for using the nerve block varied broadly, from procedures such as third molar extraction to extremely painful procedures such as post-trauma jaw manipulation. Notable differences were observed in the types of US machines used and their respective probe tip frequencies. In summary, although we attempted to maintain consistent outcome measures (pain), the methods used for assessing pain varied across individual studies.

In conclusion, the results of this systematic review demonstrate that USG IANB can effectively manage pain in adult patients undergoing certain surgical procedures, such as third molar extractions, or suffering from chronic pain conditions, such as those associated with TMD.

## Acknowledgments

The authors acknowledge the use of ChatGPT (OpenAI) to assist in drafting 2 figures (Figs. 3 and 4) related to the NIH and CARE risk of bias tools. Final content was reviewed and approved by the authors.

## Author contributions

**Conceptualization:** Daniah M. Alhazmi.

**Investigation:** Daniah M. Alhazmi.

**Methodology:** Daniah M. Alhazmi.

**Supervision:** Fatima M. Jadu.

**Validation:** Fatima M. Jadu.

**Writing – original draft:** Daniah M. Alhazmi.

**Writing – review & editing:** Fatima M. Jadu.
